# Distinct kinetics of immunoglobulin isotypes reveal early diagnosis and disease severity of COVID‐19: A 6‐month follow‐up

**DOI:** 10.1002/ctm2.342

**Published:** 2021-03-24

**Authors:** Siyang Yu, Jianghong An, Xuejiao Liao, Haiyan Wang, Fen Ma, Dapeng Li, Aimin Li, Weilong Liu, Siwei Zhang, Mingfeng Liao, Lei Liu, Juanjuan Zhao, Shaojun Xing, Lanlan Wei, Zheng Zhang

**Affiliations:** ^1^ Institute of Hepatology, National Clinical Research Center for Infectious Disease, Shenzhen Third People's Hospital; The Second Affiliated Hospital, School of Medicine Southern University of Science and Technology Shenzhen Guangdong China; ^2^ Microbiology Department Harbin Medical University Harbin Heilongjiang China; ^3^ Center Lab of Longhua Branch Shenzhen People's Hospital Shenzhen Guangdong China; ^4^ Guangdong Provincial Key Laboratory of Regional Immunity and Diseases Shenzhen University Health Science Center Shenzhen China; ^5^ Shenzhen Research Center for Communicable Disease Diagnosis and Treatment of Chinese Academy of Medical Science Shenzhen Guangdong China

To the Editor:

This study provides a comprehensive dynamic analysis of different antibody isotypes against SARS‐CoV‐2 and their relation to disease severity or early COVID‐19 diagnosis in a large patient cohort over a 6‐month follow‐up period, which will benefit the early diagnosis and assessment of disease severity for COVID‐19.

Despite ongoing studies on the kinetics of SARS‐CoV‐2‐specific antibodies,^1^, ^2^, ^3^, ^4^, ^5^ it is still insufficient for the comprehensive analysis of long‐term kinetics and durability of viral‐specific antibody isotypes and their relation to disease severity or early COVID‐19 diagnosis. In this study, we evaluated the dynamics of SARS‐CoV‐2‐specific immunoglobulins (A, G, and M) and its correlation to viral clearance and disease severity in a large cohort of COVID‐19 patients for 6 months. Besides, IgA and IgG in saliva and bronchoalveolar lavage fluid (BALF) were also assessed for the diagnosis of COVID‐19.

A total of 506 patients diagnosed with COVID‐19 basing on the World Health Organization's interim guidance (WHO 2020) were followed up for 6 months at the Shenzhen Third People's Hospital in this study. All patients were classified into asymptomatic, mild, moderate, and severe groups by the disease severity of COVID‐19 (Table 1). IgA, IgG, and IgM against the SARS‐CoV‐2 spike protein receptor‐binding domain (RBD) was measured (Chemiluminescence immunoassay kit, Beijing Wantai Biotech) in 2628 of plasma from 44 asymptomatic carriers, 29 mild, 340 moderate, and 93 severe patients (Table 1). The overall seroconversion rates of IgA, IgG, and IgM during the follow‐up period were 91.93%, 99.59%, and 61.49%, respectively (Table S1).

**TABLE 1:** Demographic and clinical characteristics of 506 COVID‐19 patients at the Third People's Hospital of Shenzhen, China

	Total	Asymptomatic	Mild	Moderate	Severe	*p*‐Value
Characteristics	*N* = 506	*n* = 44	*n* = 29	*n* = 340	*n* = 93	
Age, *n* (%)						<.0001
Median (years)	42	26	22	41	61	
0–6	18 (3.56)	5 (11.36)	2 (6.90)	11 (3.24)	0	
7–18	31 (6.13)	8 (18.18)	6 (20.69)	17 (5.00)	0	
19–45	225 (44.47)	26 (59.09)	21 (72.41)	164 (48.24)	14 (15.05)	
46–60	130 (25.69)	5 (11.36)	0	93 (27.35)	32 (34.41)	
>60	102 (20.16)	0	0	55 (16.18)	47 (50.54)	
Gender, *n* (%)						.0095
Male	249 (49.21)	18 (40.91)	12 (41.38)	159 (46.76)	60 (64.52)	
Female	257 (50.79)	26 (59.09)	17 (58.62)	181 (53.24)	33 (35.48)	
Comorbidities, *n* (%)						
None	421 (83.20)	44 (100.00)	28 (96.55)	293 (86.18)	56 (60.22)	<.0001
Hypertension	59 (11.66)	0	0	36 (10.59)	23 (24.73)	<.0001
Diabetes	21 (4.15)	0	0	9 (2.65)	12 (12.90)	<.0001
Coronary heart disease	12 (2.37)	0	0	1 (0.29)	11 (11.83)	<.0001
Epidemiological information, *n* (%)						.0194
Tourism or residence in Hubei Province	413 (81.62)	30 (68.18)	20 (68.97)	284 (83.53)	79 (84.95)	
Not been to Hubei	93 (18.38)	14 (31.82)	9 (31.03)	56 (16.47)	14 (15.05)	
Disease onset time, *n* (%)						<.0001
Early (January or earlier)	329 (65.02)	0	11 (37.93)	234 (68.82)	84 (90.32)	
Medium (February)	100 (19.76)	9 (20.45)	9 (31.03)	75 (22.06)	7 (7.53)	
Late (March or later)	77 (15.22)	35 (79.55)	9 (31.03)	31 (9.12)	2 (2.15)	
Fever, *n* (%)						<.0001
Yes	300 (59.29)	0	14 (48.28)	207 (60.88)	79 (84.95)	
No	206 (40.71)	44 (100.00)	15 (51.72)	133 (39.120	14 (15.05)	

*Note*. Categorical variables were represented as frequency (*n*) and percentage (%). Chi‐square tests or Fisher exact tests were used to compare categorical variables.

To understand the correlation between antibody responses and disease severity, the levels of viral‐specific IgA, IgG, and IgM were analyzed in four groups, and their kinetics are shown in Figures 1A–C and 2A–C. All three antibody isotypes (IgA, IgG, and IgM) peaked 1 month after disease onset (Figure 1A–C). After this peak, IgG remained at high level across patients including asymptomatic carriers but slowly declined by the sixth month after disease onset (Figure 1B). IgA also gradually declined after peak (Figure 1A). In contrast, IgM was present in serum for only 4 months (Figure 1C). These data indicated that the host antibody responses against spike protein RBD were induced in all disease severity groups and could be kept over 6 months at least after onset of illness.^6^, ^7^

**FIGURE 1:**
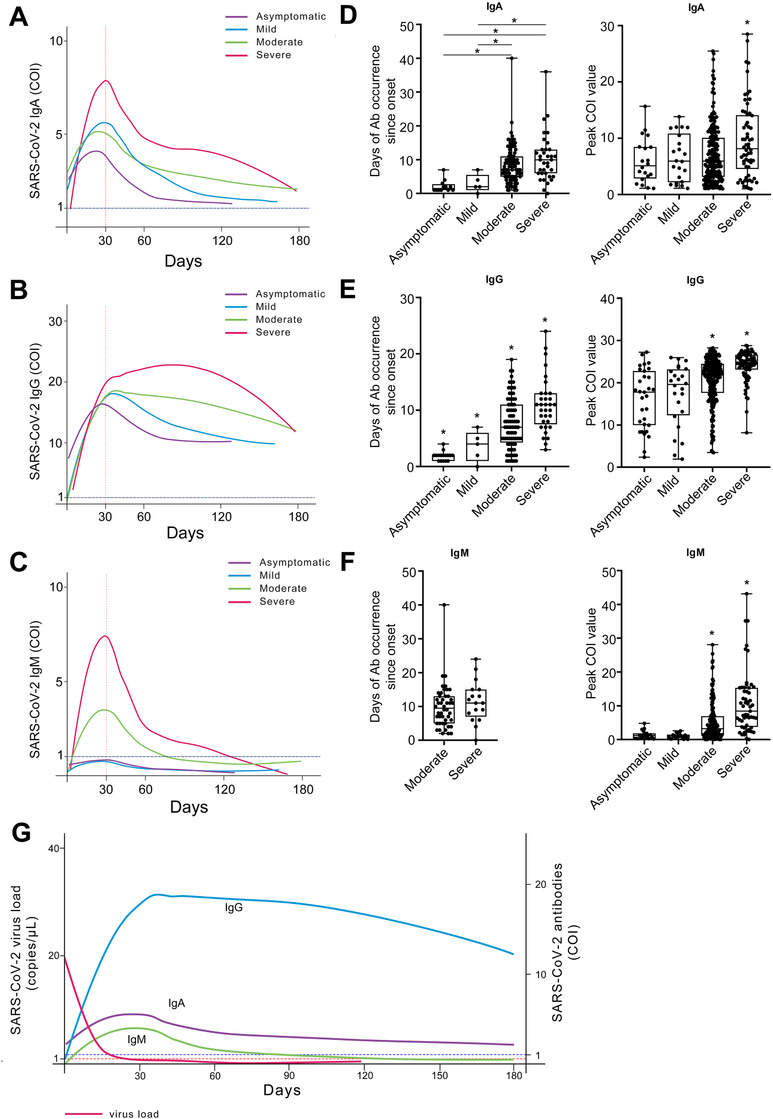
Dynamic characteristics of antibodies and virus in COVID‐19 patients after disease onset. Of all 506 patients, 9% (44 cases) were classified as asymptomatic, 6% (29 cases) mild, 67% (340 cases) moderate, and 18% (93 cases) severe. Kinetic curves of IgA (A), IgG (B), and IgM (C) grouped by clinical classification. The kinetic curves are drawn by ggplot2 package of R. The time of antibody occurrence since onset and peak cut‐off‐index (COI) value of IgA (D), IgG (E), and IgM (F) were analyzed and presented in box plots. Box plots show median, interquartile range (IQR), and range from minimum to maximum value. Kruskal–Wallis tests followed by the Mann–Whitney *U*‐test were performed to determine statistically significant differences in multiple groups. *p < .05. The kinetic curves of antibodies and SARS‐CoV‐2 virus load of all patients are drawn and merged in (G). Note: The results ≥1 COI are positive, and the results <1 COI are negative. The peak COI value and seroconversion time of antibody isotypes were analyzed based on the results of serum antibody test

**FIGURE 2:**
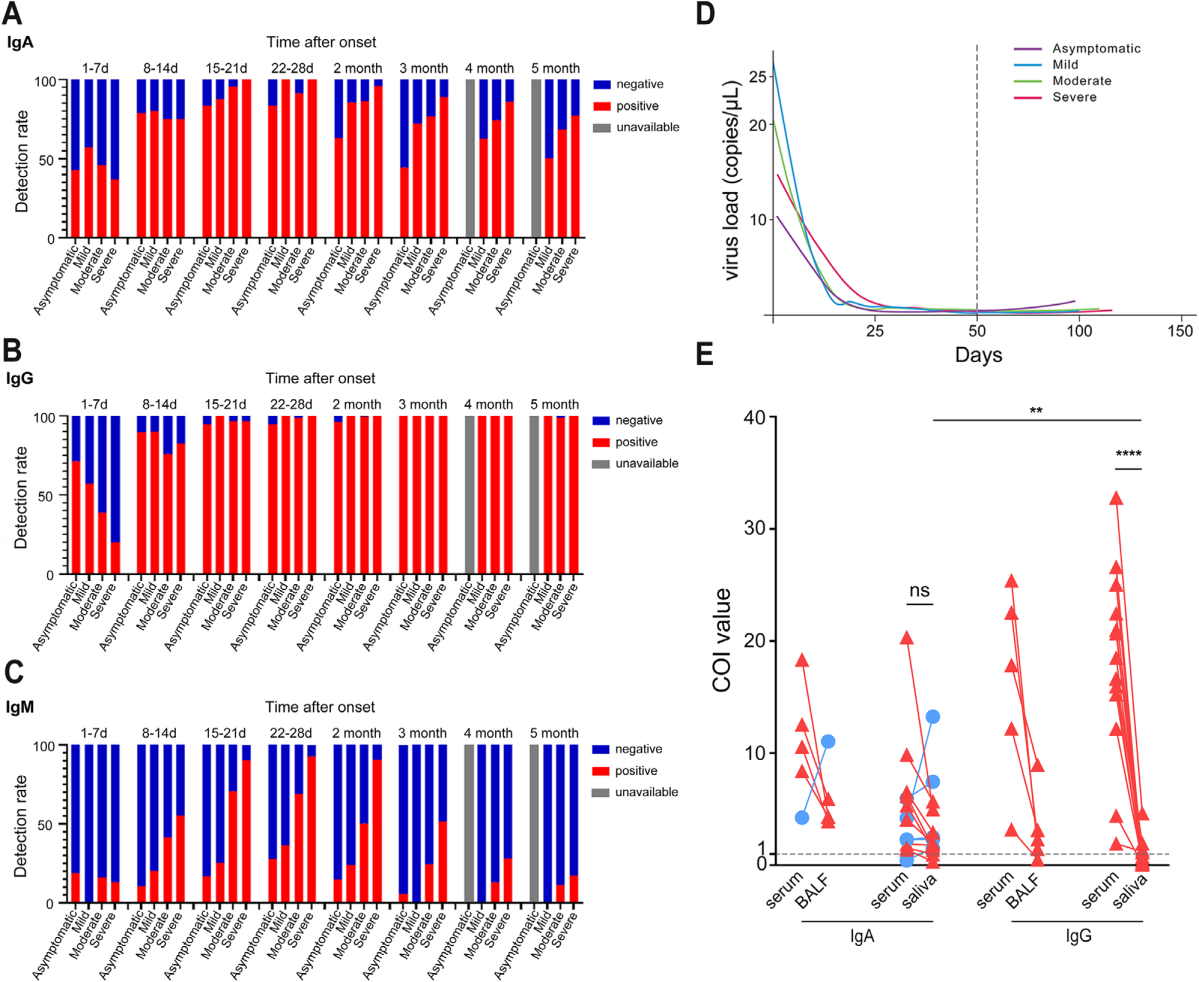
Virus load kinetic curve and antibodies detection in COVID‐19 patients. The detection rates of IgA (A), IgG (B), and IgM (C) in different phases of the disease grouped by clinical classification are shown. Kinetic curves of SARS‐CoV‐2 virus load (D) grouped by clinical classification. IgA and IgG were detected in BALF, saliva, and corresponding serum samples (E). IgA detection was performed in five BALF samples (five of five were positive) and 15 saliva samples (13/15 were positive). IgG detection was performed in five BALF samples (four of five were positive) and 15 saliva samples (five of 15 were positive). Red triangle and blue circle depict the decrease of antibody COI and the increase compared to that in serum, respectively. Paired‐sample *t*‐test was used to determine the statistically significant difference in IgA or IgG levels between serum and saliva samples, and between IgA and IgG levels in saliva samples

Interesting, IgA, IgG, and IgM levels were highest in severe COVID‐19 patients as compared to those in other groups (*p* < .05, Figure 1D–F). Notably, there were more rapid (days after onset) but less robust (peak COI) IgG responses after disease onset in asymptomatic and mild patients compared to moderate and severe patients (*p *< .05, Figure 1E). Like IgG, IgA was also present later but at higher COI levels in severe patients (Figure 1D). IgM was present at higher levels in severe COVID‐19 patients (median COI: 6.48; IQR: 2.43–14.27) compared to moderate (median COI: 1.40; IQR: 0.50–4.87), mild (median COI: 0.64; IQR: 0.22–1.34), or asymptomatic patients (median COI: 0.40; IQR: 0.27–1.19) (Figure 1F). Additionally, SARS‐CoV‐2 was cleared and undetectable 1 month after symptom onset in all groups (Figure 2D) when SARS‐CoV‐2‐specific antibody responses peaked (Figure 1G). The seroconversion time of IgA, IgG, or IgM was negatively correlated with the lowest Ct value of virus (*p* < .05 for each, Table S2) and peak levels of total Ig, IgA, IgG, and IgM were risk factors that could be used as independent predictors of disease severity (*p* < .05 for each, Table 2). These data indicated that the more rapid antibody response occurred more frequently in asymptomatic carriers, while the robustness of antibody was associated with disease severity.^8^ However, the seroconversion time of antibodies related to disease severity is controversial and needs further analysis.^2^, ^9^, ^10^

**TABLE 2:** Correlation between SARS‐CoV‐2‐specific antibodies and COVID‐19 disease severity in 506 COVID‐19 patients at the Third People's Hospital of Shenzhen, China

	Univariable analysis		Multivariable analysis^a^	
	OR (95% CI)	*p*‐Value	OR (95% CI)	*p*‐Value
First‐week seroconversion of total Ig	0.396 (0.139, 0.977)	.0583	0.403 (0.096, 1.462)	.1842
Peak total Ig	1.011 (1.008, 1.014)	<.0001	1.010 (1.006, 1.014)	<.0001
First‐week seroconversion of IgA	0.675 (0.290, 1.511)	.3475	0.686 (0.213, 2.119)	.5155
Peak IgA	1.080 (1.038, 1.123)	.0001	1.060 (1.013, 1.110)	.0119
First‐week seroconversion of IgG	0.301 (0.106, 0.742)	.0141	0.457 (0.112, 1.624)	.2419
Peak IgG	1.266 (1.182, 1.370)	<.0001	1.225 (1.131, 1.341)	<.0001
First‐week seroconversion of IgM	0.850 (0.233, 2.467)	.7816	0.783 (0.155, 3.404)	.7523
Peak IgM	1.084 (1.053, 1.119)	<.0001	1.035 (1.009, 1.068)	.0165

^a^ Adjusted for age, sex, fever, presence of hypertension, diabetes, and cardiovascular disease. Logistic regression models were used to determine independent predictors associated with the severity of COVID‐19.

To investigate whether the serological assay of different antibody isotypes could improve the early diagnostic power of COVID‐19 patients, the seroconversion rates of single or combined antibody isotypes and the detection of viral RNA in the first month after disease onset were analyzed irrespective of disease severity. In each week after disease onset, the seroconversion rate of IgA was significantly higher than IgM (Table 3 and Figure 2A). And the co‐seroconversion rate of IgA and IgG (33.11%) was higher than that of IgM and IgG (13.51%) within the first week after disease onset (Table 3), suggesting virus‐specific IgA was more sensitive than IgM for the early detection of SARS‐CoV‐2 infection. Within the first month after onset, the co‐seroconversion of IgA and IgG could improve the positive rate of serologic assay than that of IgG and IgM (93.94% vs. 77.78%). Accordingly, positive rates based on co‐seroconversion of IgA and IgG combined with viral RNA detection were higher than those of co‐seroconversion of IgG and IgM combined with the RNA assay (week 1: 83.11% vs. 70.27%; week 2: 92.67% vs. 77.33%; week 3: 97.14% vs. 87.62%; week 4: 96.97% vs. 85.86%, respectively). Next, we analyzed the distribution of IgA and IgG in BALF and saliva of COVID‐19 patients. IgA and IgG were detectable in BALF (five of five for IgA; four of five for IgG) and serum (Figure 2E). Intriguingly, the level of IgA was similar between serum and saliva (*p *> .05, Figure 2E), but the level of IgG was much lower in saliva than in serum (*p *< .0001). Moreover, IgA was the major antibody in saliva, which was much higher than IgG (*p *< .01, Figure 2E). These data suggest IgA could be more sensitive marker than IgM in the early diagnosis of COVID‐19, especially using saliva as easily collected and less‐infectious specimen in clinic.

**TABLE 3:** Positive detection rate of different SARS‐CoV‐2 virus antibodies at different time periods after disease onset among 506 COVID‐19 patients at the Third People's Hospital of Shenzhen, China

Days after onset	*n*	IgA+	IgG+	IgM+	RNA+	IgG+IgM+	IgA+IgG+	RNA+ or IgG+IgM+	RNA+ or IgA+IgG+
1–7	148	67	60	22	96	20	49	104	123
		45.27%	40.54%	14.86%	64.86%	13.51%	33.11%	70.27%	83.11%
8–14	150	114	117	64	81	64	103	116	139
		76.00%	78.00%	42.67%	54.00%	42.67%	68.67%	77.33%	92.67%
15–21	105	100	102	79	48	78	99	92	102
		95.24%	97.14%	75.24%	45.71%	74.29%	94.29%	87.62%	97.14%
22–28	99	94	98	77	26	77	93	85	96
		94.95%	98.99%	77.78%	26.26%	77.78%	93.94%	85.86%	96.97%

Abbreviations: +, positive results; IgM+IgG+, co‐seroconversion of IgM and IgG; IgA+IgG+, co‐seroconversion of IgA and IgG.

In summary, this study comprehensively analyzed the robustness and durability of SARS‐CoV‐2‐specific antibody responses and their relation to disease severity in a large patient cohort over a 6‐month follow‐up period. There were more robust IgA, IgG, and IgM responses in severe patients, more rapid but less robust IgA and IgG responses in asymptomatic and mild patients. Additionally, IgA was more likely to appear not only in serum but also in saliva during the acute phase of SARS‐CoV‐2 infection. This study therefore provides new insights for the early diagnosis and prognosis of SARS‐CoV‐2 infection and improves the understanding of the antibody response in COVID‐19.

## CONFLICT OF INTEREST

The authors declare that there is no conflict of interest.

## AUTHOR CONTRIBUTIONS

Zheng Zhang, Lanlan Wei, Shaojun Xing, Juanjuan Zhao, and Lei Liu designed the study and wrote the manuscript. Jianghong An, Xuejiao Liao, Haiyan Wang, Aimin Li, Weilong Liu, Siwei Zhang, Mingfeng Liao, and Lei Liu collected clinical specimens and clinical information. Siyang Yu and Lanlan Wei executed the experiments. Siyang Yu, Fen Ma, Dapeng Li, Haiyan Wang, and Xuejiao Liao analyzed the data. All the authors approved the final version.

## Supporting information


**Supporting Table S1** The seroconversion rate of SARS‐CoV‐2‐specific IgA, IgG, and IgM during the follow‐up period in all COVID‐19 patients at the Third People's Hospital of Shenzhen, China
**Supporting Table S2** Correlation between SARS‐CoV‐2 specific antibodies and viral nucleic acid in 506 COVID‐19 patients at the Third People's Hospital of Shenzhen, China
