# Supplementary material for: Distinct kinetics of immunoglobulin isotypes reveal early diagnosis and disease severity of COVID‐19: A 6‐month follow‐up
Source: Clin Transl Med. 2021 Mar 24;11(3):e342. doi: 10.1002/ctm2.342 (PMC7989708; doi:10.1002/ctm2.342)
Supplement: Supplementary file 1 — Supporting Table S1 The seroconversion rate of SARS‐CoV‐2‐specific IgA, IgG, and IgM during the follow‐up period in all COVID‐19 patients at the Third People's Hospital of Shenzhen, China Supporting Table S2 Correlation between SARS‐CoV‐2 specific antibodies and viral nucleic acid in 506 COVID‐19 patients at the Third People's Hospital of Shenzhen, China [file CTM2-11-e342-s001.docx]

**Supplemental Tables**

**Supplemental Table 1.** The seroconversion rate of SARS-CoV-2 specific IgA, IgG, and IgM during the follow-up period in all COVID-19 patients at the Third People’s Hospital of Shenzhen, China

| Weeks or Months after Disease onset | IgA | IgG | IgM |
| --- | --- | --- | --- |
| Week 1 | 44.30% | 40.51% | 15.19% |
| Week 2 | 75.84% | 79.78% | 39.89% |
| Week 3 | 94.56% | 96.60% | 65.75% |
| Week 4 | 93.66% | 98.59% | 68.31% |
| Month 2 | 86.60% | 99.50% | 54.09% |
| Month 3 | 77.41% | 100.00% | 28.01% |
| Month 4 | 77.11% | 100.00% | 16.06% |
| Month 5 | 68.85% | 99.18% | 12.30% |
| Month 6 | 73.91% | 100.00% | 4.35% |
| Overall Seroconversion Rate | 91.93% | 99.59% | 61.49% |

**Supplemental Table 2.** Correlation between SARS-CoV-2 specific antibodies and viral nucleic acid in 506 COVID-19 patients at the Third People’s Hospital of Shenzhen, China.

|  | IgA | | | | | |  |
| --- | --- | --- | --- | --- | --- | --- | --- |
|  | Peak COI | |  | SC time | |  |  |
|  | r | *p* value | | r | *p* value | |  |
| First Ct value | 0.04036 | 0.5704 |  | -0.1041 | 0.3078 |  |  |
| Lowest Ct value | -0.003595 | 0.96 |  | -0.2507 | 0.0128 | ***** |  |
| Shedding duration | 0.1174 | 0.1086 |  | 0.04696 | 0.6621 |  |  |
|  | IgG | | | | | |  |
|  | Peak COI | |  | SC time | |  |  |
|  | r | *p* value | | r | *p* value | |  |
| First Ct value | 0.08931 | 0.1761 |  | -0.1381 | 0.2132 |  |  |
| Lowest Ct value | 0.08931 | 0.5255 |  | -0.2253 | 0.0393 | ***** |  |
| Shedding duration | -0.03789 | 0.5806 |  | 0.07068 | 0.5496 |  |  |
|  | IgM | | | | | |  |
|  | Peak COI | |  | SC time | |  |  |
|  | r | *p* value | | r | *p* value | |  |
| First Ct value | 0.01448 | 0.8474 |  | -0.2565 | 0.089 |  |  |
| Lowest Ct value | -0.08772 | 0.2429 |  | -0.4476 | 0.002 | ***** |  |
| Shedding duration | 0.1219 | 0.1543 |  | -0.02588 | 0.8724 |  |  |
|  |  |  |  |  |  |  |  |

Note: SC: seroconversion time. * means *p* < 0.05. Pearson’s correlation coefficient was used to assess the relationship between antibodies and COVID-19 severity.
